# Molecular Mimicry between Chikungunya Virus and Host Components: A Possible Mechanism for the Arthritic Manifestations

**DOI:** 10.1371/journal.pntd.0005238

**Published:** 2017-01-26

**Authors:** Vijayalakshmi Reddy, Anita Desai, Shankar Susarla Krishna, Ravi Vasanthapuram

**Affiliations:** 1 Department of Neurovirology, National Institute of Mental Health and Neurosciences, Bangalore, Karnataka, India; 2 Department of Neuropathology, National Institute of Mental Health and Neurosciences, Bangalore, Karnataka, India; Stanford University School of Medicine, UNITED STATES

## Abstract

**Background:**

Chikungunya virus (CHIKV), a reemerging pathogen causes a self limited illness characterized by fever, headache, myalgia and arthralgia. However, 10–20% affected individuals develop persistent arthralgia which contributes to considerable morbidity. The exact molecular mechanisms underlying these manifestations are not well understood. The present study investigated the possible occurrence of molecular mimicry between CHIKV E1 glycoprotein and host human components.

**Methodology:**

Bioinformatic tools were used to identify peptides of CHIKV E1 exhibiting similarity to host components. Two peptides (A&B) were identified using several bioinformatic tools, synthesised and used to validate the results obtained *in silico*. An ELISA was designed to assess the immunoreactivity of serum samples from CHIKV patients to these peptides. Further, experiments were conducted in a C57BL/6J experimental mouse model to investigate if peptide A and peptide B were indeed capable of inducing pathology.

**Findings:**

The serum samples showed reactivity of varying degrees, indicating that these peptides are indeed being recognized by the host immune system during CHIKV infection. Further, these peptides when injected into C57BL/6J mice were able to induce significant inflammation in the muscles of C57BL/6J mice, similar to that observed in animals that were injected with CHIKV alone. Additionally, animals that were primed initially with CHIKV followed by a subsequent injection of the CHIKV peptides exhibited enhanced inflammatory pathology in the skeletal muscles as compared to animals that were injected with peptides or virus alone. Collectively these observations validate the hypothesis that molecular mimicry between CHIKV E1 protein and host proteins does contribute to pathology in CHIKV infection.

## Author Summary

The outcome of Chikungunya virus infection is usually benign but persistent arthritis has been reported in 10–20% of patients after Chikungunya fever. However, some reports have suggested that similarity between host proteins and viral proteins (molecular mimicry) leads to immune mediated damage. However, this has not been proved conclusively. Therefore, this study was undertaken to identify if molecular mimicry exists between CHIKV and host components. Using various bioinformatics tools we identified common sequences and structural homology between glycoprotein of the virus and two human host tissue proteins- HLA-B27 molecule and a domain of complement C3. Two peptides having homology to these human tissue components were synthesized. These peptides were recognized by antibodies present in serum of CHIKV patients. Experiments were conducted to investigate if the peptides were capable of inducing pathology in an experimental C57BL/6J mouse model. Both the peptides on their own were able to induce significant inflammation in the muscles of C57BL/6J mice similar to that observed in animals that were injected with CHIKV alone. Additionally, animals that were injected initially with CHIKV followed by a subsequent injection of the two CHIKV peptides exhibited increased pathology as compared to animals that were injected with peptides or virus alone.

## Introduction

Chikungunya fever is caused by a arbovirus belonging to Family *Togaviridae* and Genus *Alphavirus*. CHIKV is positive sense RNA virus with about 11.8 kb long genome. The prevalence of CHIKV has increased globally. It caused massive outbreaks when it re- emerged in 2005 in French Reunion islands where it affected about 33% of the total population. CHIKV outbreaks also occurred in India during the same period, southern states in India recorded a total of 1.3 million cases [1,2]. Chikungunya fever is characterized by fever, headache, myalgia and arthralgia. Though Chikungunya is a self limiting illness [3], a small proportion of 10–20% of affected individuals develop persistent arthralgia. The risk factors associated with the development of persistent arthralgia include older age of patients (>40 years) and pre existing rheumatic problems. However, the precise molecular mechanisms of pathogenesis that lead to the development of these complications are poorly understood. Experimental evidence of CHIKV persistence in macrophages of *Macaca species* has been demonstrated [4] and it has been suggested as one of the factors contributing to residual arthralgia. Although, molecular mimicry as the cause of prolonged joint manifestations had not been proved conclusively in Chikungunya infection, there are reports which suggest that such a phenomenon might be operational. Therefore, in this study we investigated the possible occurrence of molecular mimicry between CHIKV E1 and host components using a three pronged strategy: (i) identification of homologous regions between CHIKV proteins and host tissue components using bioinformatics tools, (ii) establishing cross reactivity between serum samples obtained from CHIKV infected patients and peptides exhibiting molecular mimicry and (iii) validating the ability of the cross reactive peptides in inducing joint and muscle pathology in a mouse model. We demonstrate the occurrence of molecular mimicry between CHIKV envelope glycoprotein (E1) and the host components.

## Methods

### Virus

A clinical isolate of CHIKV (Chikungunya virus strain DRDE-06; GenBank accession number: EF210157.2) was used for all the *in vivo* experiments in this study. The bioinformatics related work was carried out using the CHIKV E1 protein sequence from the prototype strain CHIKV S27 available in the SWISS PROT (ID:Q8JUX5). Further, a multiple sequence alignment of the E1 glycoprotein of DRDE-06 sequences and CHIKV S27 revealed a 98% homology between the two strains.

### Peptides

CHIKV peptides were custom synthesised from commercial sources (Hysel Pvt Ltd., India) and obtained as a lyophilised powder. The non-specific peptide was a gift from XCyton diagnostics private Ltd, Bangalore, India.

### Antibodies

Rabbit anti-human polyclonal-HRP conjugate was procured from Dako, Denmark, while Goat anti-mouse IgG-HRP was obtained from Genei, Bangalore.

### Ethics statements

All work related to animals was conducted with good animal practice defined by committee for the purpose of control and supervision of experiments of animals. The use of animals was approved by the institutional animal ethics committee (IAEC) of NIMHANS (Approval reference no: AEC/41/222(B)/NV dated 05.10.2010). The animals were housed in cages maintained in hygienic conditions with good ventilation, in a room maintaining the usual day and night cycle. The animals used for the experiments were euthanized by cervical dislocation and animal ethics were strictly adhered to at all times, while bleeding and sacrificing the animals.

The use of human samples for the study was approved by was approved by institute ethics committee at NIMHANS (Approval reference no: NIMHANS/68th IEC/2010) which adheres to the ethical guidelines for biomedical research on human participants developed by the Indian Council for Medical research (ICMR). Written informed consent was obtained from all the subjects themselves in the study.

### Animals

C57BL/6J strain of mice were obtained from NIMHANS Central animal research facility and used in the study. Eight day old mouse pups were procured from the animal facility along with the mother and the mouse pups were used for the experiments.

### Clinical samples

The human samples used in this study were received at the Department of Neurovirology, National Institute of Mental Health and Neurosciences (NIMHANS), which is one of the twelve designated national apex laboratories for the diagnosis of Chikungunya in India. All the subjects enrolled in the study presented to the hospital/clinics with fever, joint pain, rash, myalgia, conjunctival redness, and headache. Additionally, the prevalence and local outbreaks in the region aided in making a clinical diagnosis of Chikungunya fever. Blood samples (3–5 ml clotted blood) were collected from thirty six subjects, serum separated and stored in aliquots at -70°C until all the tests were performed. The CHIKV infection was confirmed by detection of CHIKV specific IgM antibodies using an ELISA (National Institute Virology, Pune) and/or CHIKV RNA by TaqMan real time PCR targeting the NSP4 region [5]. Serum samples collected from 31 healthy individuals served as controls.

### Concentration and purification of CHIKV

CHIKV was grown in C6/36 cell line and infectious fluid was harvested. CHIKV infected C6/36 fluid was centrifuged at 10,000 rpm for 20minutes to remove debris and NaCl was added to the supernatant to obtain a final concentration of 0.5 molar. Subsequently, polyethylene glycol was added to the mixture to obtain a final concentration of 10% (w/v) and the suspension stirred on ice bath for 20 minutes. The mixture was incubated overnight at 4°C, and centrifuged at 3000 rpm for 30 minutes to obtain the virus rich precipitate. The precipitate was dissolved in 1/100th of original infected cell culture fluid volume using GTNE buffer. CHIKV was purified using a discontinuous sucrose gradient method. Briefly, 5ml of 20% sucrose (w/v) in GTNE buffer was carefully overlaid onto 2.5ml of 50% (w/v) sucrose. Subsequently, 2.5ml of CHIKV obtained after PEG concentration was overlaid onto the discontinuous sucrose gradient and centrifuged at 28,000 rpm for 2 hours at 4°C using a ultracentrifuge (Beckman Coulter, USA). The band at the inter-phase was collected and re-suspended in 10–12 volumes of PBS (pH7.2) and centrifuged at 28,000 rpm for 2 hours to obtain a purified virus pellet. The pellet was dissolved in 1ml of fresh PBS and frozen in small aliquots at -70°C.

### Bioinformatic approach to determining molecular mimicry

The complete genome sequence of a prototype CHIKV S27 belonging to African genotype was obtained from the Gen Bank. The sequence of CHIKV E1 glycoprotein was obtained from SWISSPROT (Q8JUX5). This sequence was uploaded into Immune Epitope Database and Analysis Resource (IEDB) server available at http://www.immuneepitope.org/. The server predicts the antigenic determinants using five different algorithms—Chou and Fasman beta turn prediction, Emini surface accessibility prediction, Karplus and Schulz flexibility prediction, Kolaskar and Tongoankar antigenicity prediction, Parker hydrophilicity prediction. The antigenic peptides from E1 region were deduced after considering hydrophilicity, surface probability, chain flexibility and secondary structure antigenic index both as text and graphs. The results obtained from the server were combined to construct a graph using MS- EXCEL, which in turn yielded putative epitopic regions of CHIKV E1 glycoprotein. The peaks with antigenic propensity, surface accessibility, flexibility, hydrophilicity and beta turns were considered. The results obtained through IEDB were further confirmed using additional server- European Molecular Biology Open Software Suite (EMBOSS) available at http://liv.bmc.uu.se/cgi-bin/emboss/antigenic. The results obtained using the Chou and Fasman criteria for beta turn prediction was also verified using COUDES server. The existence of sequence similarity between CHIKV E1 glycoprotein and Human HLA-B27 was investigated using BLAST. The existence of structural similarity between the CHIKV E1 glycoprotein and human host components were determined by using BioXGEM server and number of hits obtained in the non-redundant protein database (nrPDB) were limited to first 100 in the output. Multiple sequence alignment of E1 glycoprotein sequences of Alphaviruses- CHIKV, ONNV, RRV, SFV, and EEEV was done using CLUSTALW available at http://www.ebi.ac.uk/Tools/clustalw2/index.html.

### Immunoreactivity of serum samples obtained from patients infected with CHIKV to the peptides derived from CHIKV glycoprotein E1

The optimal concentration of the peptide to be coated onto the ELISA plate was predetermined in an initial experiment and was found to be 25μg/well. The peptides were coated onto the ELISA microwells using carbonate buffer and incubated overnight at 4°C. The plate was washed three times with phosphate buffered saline with tween (PBST) and quenched using 1% skimmed milk powder in PBST for one hour at 37°C. The plates were washed with PBST and reacted with 100μl of serum samples (1:100 dilution in PBS containing 0.25% triton-X 100) obtained from patients infected with CHIKV (n = 36) as well as serum samples obtained from control subjects (n = 31). The samples were incubated for 90 minutes at 37°C, followed by five washes with 1X PBST. Subsequently, rabbit polyclonal anti-human antibodies tagged with HRP (Dako, Denmark) was diluted 1:1000 and 100μl was added to each well and the plate was incubated at room temperature for 90 minutes. The plate was washed five times with PBST and 100μl of the TMB solution was added and incubated in the dark for 10 minutes. The reaction was stopped by the addition 4N sulphuric acid and the OD values were read at 492nm using ELISA reader (Thermo scientific, USA).

### Mouse experiments to evaluate the role of CHIKV peptides in causing tissue damage by molecular mimicry

Eight day old C57BL/6J pups were procured along with the mother and the pups were used for the *in vivo* experiments. They were assigned to nine different groups with each group comprising of 6 pups as shown in Table 1.

**Table 1:** Details of the experimental animals used to study the pathology induced by injection of CHIKV, CHIKV specific peptides and non specific peptide either alone or in combination.

EXPERIMENTAL GROUPS OF C57BL/6J MICE (n = 6 per group)	NATURE OF INOCULUM
Group 1: Virus control	CHIKV alone (10,000PFU/50μl)
Group 2: Mock infected control	EMEM alone
Group 3: CHIKV Peptide A control	Two doses of peptide A (50μg/dose) emulsified in Freund's incomplete adjuvant and injected on day 0 and day 5
Group 4: CHIKV Peptide B control	Two doses of peptide B (50μg/dose) emulsified in Freund's incomplete adjuvant and injected on day 0 and day 5
Group 5: Non specific peptide control	Two doses of non specific peptide (50μg/dose) emulsified in Freund's incomplete adjuvant and injected on day 0 and day 5
Group 6: Adjuvant (No peptide) control	EMEM followed by sterile 1X PBS emulsified in Fruend's incomplete adjuvant
Group 7: CHIKV + CHIKV Peptide A	CHIKV(10,000PFU/50μl) on day 0, followed by peptide A(50μg/dose) emulsified in Fruend's incomplete adjuvant on day 5
Group 8: CHIKV+CHIKV Peptide B	CHIKV (10,000PFU/50μl) followed by peptide B (50μg/dose) emulsified in Fruend's incomplete adjuvant on day 5
Group 9: CHIKV + Non specific peptide	CHIKV(10,000PFU/50μl) followed by non specific peptide (50μg/dose) emulsified in Fruend's incomplete adjuvant on day 5

Prior to determining the role of peptides in the possible enhancement of pathology related to CHIKV infection, the pathological changes induced by the CHIKV in C57BL/6J mice were studied (Group 1). For this purpose, CHIKV (10^5^ PFU/50μl) was inoculated into the foot pad of 8 day old mice. The control group of mice (Group 2) received equal volume (50 μl) of Eagles Minimum Essential Medium (EMEM). The mice were kept under observation for 12 days only post infection. At the end of the observation period, blood was collected from the mice through retro orbital plexus bleeding, and the mice euthanized to obtain the following organs- brain, thymus, heart, lungs, spleen, liver, kidneys, upper limbs and lower limbs. For histopathological studies the tissues were fixed in 4% paraformaldehyde, while for PCR the tissues were collected in EMEM. CHIKV infection was confirmed by two methods- presence of CHIKV specific IgG antibodies in the serum and detection of CHIKV nucleic acid in the serum and harvested tissues using TaqMan real- time PCR [5].

Eight day old pups in Group 3, 4 and 5 were injected with two doses (50μg /dose) of CHIKV Peptide A, CHIKV Peptide B and Non-specific peptide respectively on day 0 and day 5. The peptides were reconstituted in sterile 1X PBS (pH 7.2). Equal volumes of peptide solution and Freund’s incomplete adjuvant were mixed and emulsified to obtain water in oil emulsion. The emulsion was stored at -70°C until use. In all these three groups blood was collected 12^th^ day post inoculation by retro orbital bleeding and fresh tissues were harvested and processed for PCR and paraformaldehyde fixed tissues for histopathology. Mice in Group 6 were injected with EMEM alone followed by PBS emulsified in Freund’s incomplete adjuvant 5 days post infection with CHIKV.

C57BL/6J mice in Group 7, 8 and 9 were injected with CHIKV (10^5^ PFU/50 μl) followed by 50μg of the CHIKV Peptide A, CHIKV Peptide B and Non-specific peptide respectively, on 5^th^ day post CHIKV inoculation through the same route at the same site. In these groups of mice the blood was collected and tissues harvested on 12^th^ day post infection, and processed for PCR and histopathological examination.

### Detection of anti CHIKV IgG antibodies in the serum of C57BL/6J mice

Serum was separated from the blood and stored at -70°C. Polystyrene ELISA microwells (Nunc, Denmark) were coated with purified CHIKV in carbonate buffer at a concentration of 1μg/well diluted in carbonate buffer (Appendix I). The plate was incubated overnight at 4°C, washed thrice with PBST and quenched using 1X PBST containing 1% skimmed milk powder. Serum samples were diluted 1:100 in PBST and 100μl added to the wells and incubated at 37°C for 1 hour followed by washing for five times with PBST. Subsequently, 100 μl of a 1:5000 dilution of Goat anti-mouse IgG conjugated with HRP (Genei, Bangalore) was added to the wells, incubated at 37°C for 1 hour followed by washing for 5 times with PBST. Finally, 100μl of the substrate solution (TMB) was added to all the wells and incubated in the dark for 10 minutes. The reaction was stopped by the addition 4N sulphuric acid. The OD values were read at 450nm using an ELISA reader (Thermo scientific, China).

### Detection of CHIKV RNA in the harvested tissues by TaqMan real time PCR

The tissues collected in EMEM were homogenised using a motorised hand held homogeniser. The homogenates were spun at 8,000 rpm for 10 minutes at 4°C. The supernatants were collected, 200 μl of the supernatant was used for RNA extraction using QIAmp viral RNA extraction kit (Qiagen, Germany). The eluted RNA was converted to cDNA using high capacity reverse transcription kit (ABI, USA). The cDNA was stored at -20°C until tested. Similarly whole blood RNA extraction kit was used to extract RNA from blood and converted to cDNA which was used in the TaqMan real time PCR as described earlier [5].

### Histopathological study to evaluate the tissue response to injection of virus and peptides by Haematoxylin and Eosin (H&E) staining

All tissues were fixed in paraformaldehyde and embedded in paraffin for processing and 4 μm thick sections obtained were mounted on selin coated glass slides. Subsequently, the tissue sections were de-paraffinised with two changes of xylene and rehydrated in absolute alcohol followed by washing briefly in tap water. Staining of the sections was carried out using Harris haemotoxylin for 5–8 minutes, followed by washing under running tap water for 5 minutes and differentiated in 1% acid alcohol for 30 seconds. The sections were subsequently washed under running tap water for 1 minute, treated with bluing saturated lithium carbonate solution for 30–60 seconds and washed under tap water for 1 minute. Counter staining of sections was carried out using eosin-phloxine solution for 1 minute followed by dehydration in 95% alcohol and absolute alcohol for 5 minutes each. The sections were finally immersed in xylene twice for 2 minutes each for clearing and then mounted with DPX.

## Results

### Bioinformatics approach to deducing molecular mimicry

**Fig 1:**
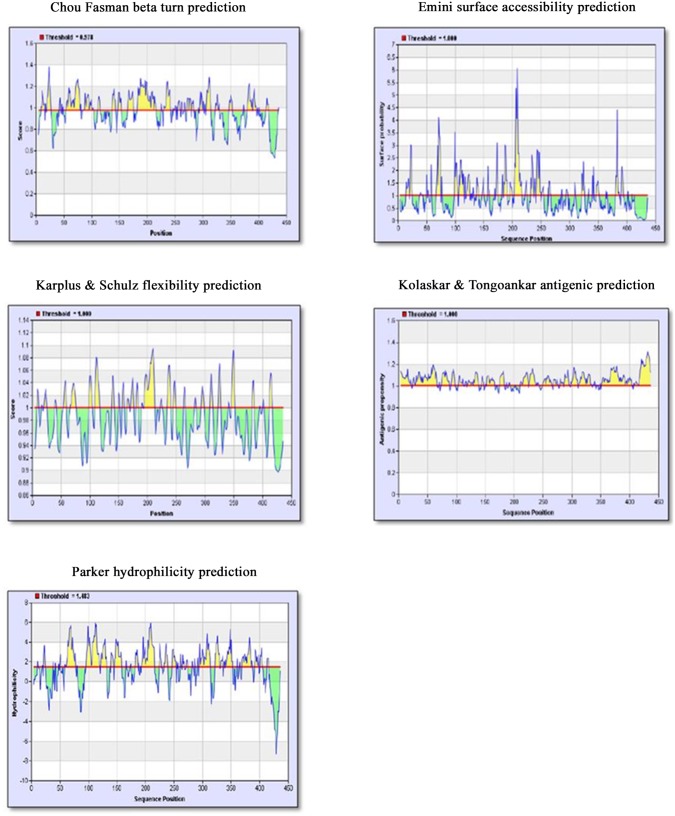
Results of five algorithms used in IEDB server to predict the immunodominant regions of CHIKV E1 glycoprotein. X-axis represents the position of amino acids of the E1 glycoprotein and Y-axis depicts the score for each algorithm. The red line indicates the threshold used by the algorithm to screen the low scored regions. The yellow peaks represent the stretch of amino acids that have the ability to satisfy the criteria being studied in the respective algorithm.

As described in the materials and methods section, the sequence of the African prototype of CHIKV S27 EI glycoprotein (Q8JUX5) was obtained from the SWISSPROT database and subjected to immune epitope analysis using five algorithms in IEDB and EMBOSS programs. The results are depicted in Fig 1. The scores obtained for the five algorithms were uploaded into an MS Excel sheet to generate a combined graph which yielded the putative epitopic regions of E1 glycoprotein of CHIKV. Analysis of the peaks in the graph with respect to antigenic propensity, surface accessibility, flexibility, hydrophilicity and B turns enabled prediction of the following epitopic regions:

Peptide A: 201 **GDIQSRTPESKDVYANTQLV** 220
Peptide B: 318 **IKYAVSKKGKCAVHSMTNAV** 338
Peptide C: 65 **TAECKDKNLPDYSCKVFTGV** 85
Peptide D: 102 **QLSEAHVEKSESCKTEFASAY** 122

**Fig 2:**
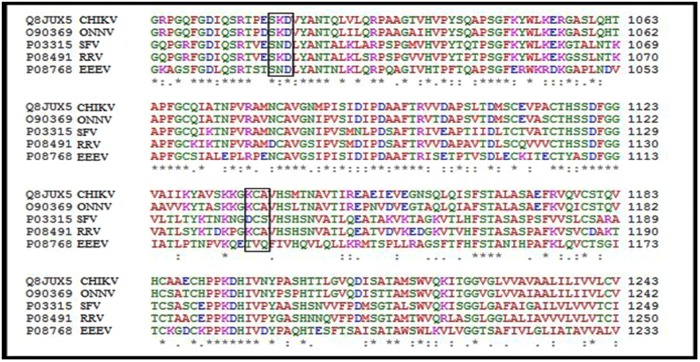
Results of alignment of alphavirus E1 glycoprotein. Alignment of amino acid sequences of E1 gylcoprotein of alphaviruses using CLUSTAL W.CHIKV, ONNV, RRV represent the arthritogenic subgroup, while SFV and EEEV represent encephalitogenic subgroup. Note the Motif SKD and KCA were present in arthritogenic alphaviruses and not in encephalitogenic alphaviruses.

These regions of CHIKV E1 glycoprotein satisfy all the criteria necessary for a given peptide to be considered immunogenic and capable of eliciting an immune response in the host system. Subsequently multiple sequence alignment of E1 glycoprotein of Alphaviruses- CHIKV, Onyong Onyong Virus (ONNV), Ross River Virus (RRV), Semiliki Forest Virus (SFV), and Eastern Equine Encephalitis Virus (EEEV) was done through CLUSTALW. The results of the alignment are depicted in Fig 2. As evident from the figure, the alignment revealed two motifs **SKD** and **KCA** present only in arthritogenic Alphaviruses (CHIKV,ONNV, RRV) and not in encephalitogenic Alphaviruses (SFV, EEEV). Furthermore, the amino acid sequences SKD and KCA were present in the immunodominant peptides 1 and 2 deduced from E1 glycoprotein respectively. All further experiments using human serum samples and mouse models were therefore restricted only to these two peptides.

**Fig 3:**
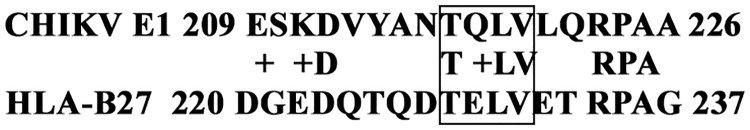
Results of alignment between HLA-B27 and CHIKV E1 glycoprotein. An alignment of amino acid sequences between alpha chain of HLA-B27 and CHIKV E1 glycoprotein. Note: An amino acid similarity (Boxed region) between the two proteins was detected by BLAST. The highlighted area shows the regions where the overlap of the amino acids occurs between CHIKV E1 glycoprotein and HLA-B27.

**Fig 4:**
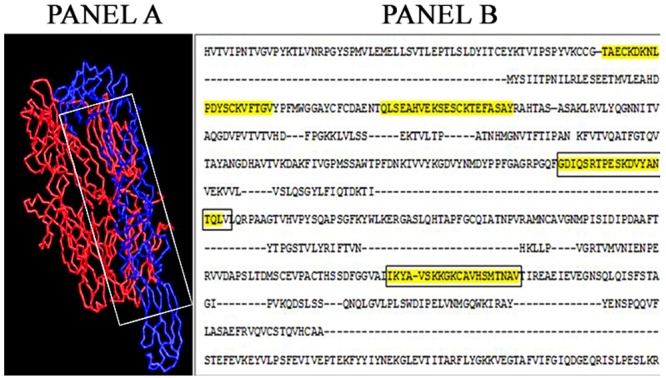
Results of amino acid alignment between C3 component of the complement and CHIKV E1 glycoprotein obtained from BioXGEM blast. Panel A shows the 3D alignment of two proteins, while panel B represents the actual areas where the structural similarity occurs. In panel A complement component C3 is depicted in red colour and CHIKV E1 glycoprotein in blue. The boxed area depicts the shared structural domains between the two proteins. In panel B the highlighted areas in the alignment indicate the areas of structural homology between C3 component of complement and CHIKV E1 glycoprotein. Further, the areas in the box indicate the sequence which is also present in the antigenic peptides (Peptide A and Peptide B)

The sequence similarity between the CHIKV E1 glycoprotein and HLA- B27 was determined using BLAST. The alpha chain of HLA-B27 molecule shared a partial homology from the stretch ranging from 216–220 of CHIKV E1 glycoprotein as well as the immunodominant region of Peptide A (Fig 3). The output obtained through the BioXGEM 3D BLAST performed on the CHIKV E1 was limited to first 100 hits. The output of the BLAST was further analyzed and limited only to human proteins (Table 2). Further analysis of these human proteins was limited to those that are known to contribute to the inflammation and arthritic pathology. Amongst these, six human proteins- Human complement component 3, complement component 5 [6], fibronectin [7], kelch like protein [8], mast/stem cell growth receptor [9] and beta arrestin 1 [10] which exhibited maximum similarity to CHIKV EI protein were only considered for further analysis. Prominent among these six were complement proteins C3 and C5. When the structural similarity between the E1 glycoprotein and complement component C3 was analyzed, the sequence of amino acids in the region of CHIKV E1 glycoprotein which shared homology with complement component C3 were also present in Peptide A and Peptide B (Fig 4).

**Table 2:** List of human proteins obtained in the BioXGEM blast which share structural homology with CHIKV E1 glycoprotein. The output was limited to 100 proteins only and all the human proteins in these were shortlisted and the most probable proteins that might contribute to the inflammation and arthritic pathology were considered.

Protein ID	Name of the protein
3EI4:E	hsDDB1-hsDDB2 complex—DNA damage binding proteins
**2A73:A**	**Human Complement Component C3**
**1FNF:A**	**Fibronectin (cell adhesion protein)**
1IGY: D	Intact IgG monoclonal antibody
**3II7:A**	**Kelch like protein**
**3GC3:A**	**Beta arrestin 1 (adaptor protein in trafficking, binds to clathrin)**
**2E9W:A**	**Mast/stem cell growth factor receptor**
**3CU7:A**	**Complement protein 5 (P.S: C3 & C5 adopt similar core structure)**
2JK4:A	Voltage dependent anion channel protein 1 (mitochondrial porin)
2FAU:A	Vacuolar protein sorting 26 (has arrestin fold)
1VYH:S	Platelet activating factor acetyl hydrolase IB alpha subunit
1K8K:C	ARP 2/3 complex 41 (initiates actin polymerisation)
3BPN:C	Interleukin-13 receptor alpha-1 chain
1FLR:H	Mouse– 4-4-2- Fab fragment
2W18:A	C terminal WD40 domain of human PALB2 (partner and localiser of BRCA2)
2V5Y:A	Receptor protein tyrosine phosphatase
2ARJ:H YTS 105.18	Antigen binding region Heavy chain (YTS 105.18, which is commonly used in the blockade of CD8+ T-cell activation in response to peptide antigen, is specific for mouse CD8alpha)
2WJS:A	Mouse—laminin subunit alpha (in basement membrane assembly and function)
2R51:A	Mouse—Vacuolar protein sorting-associated protein 26B
2OM5:A	Contactin 2 (neural cell adhesion)—consists of six immunoglobulin-like and four fibronectin III-like domains and is anchored to the membrane by glycosylphosphatidylinositol

Note: The proteins highlighted in bold font indicates the human proteins in the list implicated either in inflammation or arthritic pathology.

### Clinical features

All the patients in the study were from the state of Karnataka, South India. Among the 36 patients with confirmed CHIKV infection, 17 were females and 19 were males. The mean age of the patients was 40.64 years. All the patients whose samples were used in the study presented with fever, while 28(78%) had arthralgia, 30(83%) had myalgia, 20(56%) had complained of headache. Rash and gastrointestinal symptoms were seen in 14(39%) and 10(28%) of the patients each, and conjunctival redness was seen in 1(3%) of the patients. Amongst 3/36 patients (8%) persistent arthralgia was reported at 12 weeks after onset of initial symptoms. Hence follow up samples blood samples could be collected from these three patients at 12 weeks. In all other patients no follow up samples could be collected.

### Cross reactivity of serum samples obtained from CHIKV infected patients with peptides

**Fig 5:**
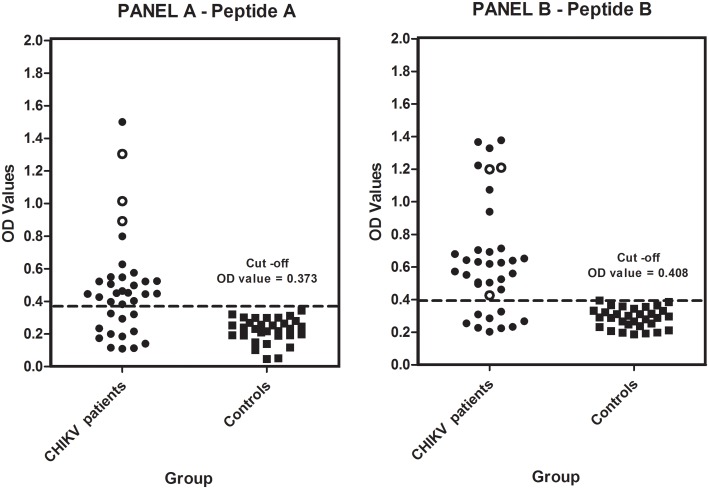
Comparison of OD values obtained in ELISA with serum samples obtained from confirmed CHIKV patients and controls using the two peptides (A and B). The X-axis represents the groups tested, while the Y-axis represents the OD Values obtained in ELISA. The horizontal line within the scatter graph represents the cut-off value obtained with respective peptides. Panel A represents OD values obtained on reacting serum samples from confirmed CHIKV patients with Peptide A while Panel B represents OD values obtained on reacting serum samples from confirmed CHIKV patients with Peptide B. Open circles (O) represent OD values obtained with Chikungunya patients (n = 3) who presented with persistent arthralgia 12 weeks after initial onset of symptoms. Peptide A shares sequence homology with HLA-B27 and shares structural homology with C3. Peptide B shows structural similarity to C3. Further, from the scatter graphs (Panel A and Panel B), it can be observed that 24/36 (66.66%) and 27/36(75%) serum samples have reacted with the peptide A and peptide B (OD values above cut off) respectively from the CHIKV confirmed group as compared to the healthy control group. Note: All the three patients with persistent arthralgia showed high OD values against Peptide A (open circles) while only 2 of these 3 showed high OD values against Peptide B.

A sample was considered to be positive in the peptide ELISA if it had an OD value equal to or greater than that of the cut-off value. The cut off value for each of the peptides was calculated by using OD values obtained with sera of healthy control subjects (n = 31) using the formula Mean OD of control samples + 2SD. The cut off OD value for Peptide A was 0.373 and for Peptide B it was 0.408. Amongst the 36 samples obtained from confirmed CHIKV patients, 24 (66.66%) showed reactivity to the peptide A and 27 (75%) to peptide B (Fig 5). The OD values of CHIKV positive samples towards these peptides ranged from 1.501 to 0.11 for Peptide A, while it varied from 1.378 to 0.203 for Peptide B.

### Histopathological features of the tissues injected with CHIKV and peptides

These experiments were carried out to determine the possible synergistic role of peptides in the enhancement of pathology in CHIKV infection: All the mice were confirmed to have CHIKV infection by the detection of anti CHIKV antibodies in the serum by ELISA. The cut-off in the ELISA was determined using the mean + 2SD OD values of serum samples obtained from uninfected control mice and it was 0.253. The OD values of serum samples from all the infected animals were found to be > 0.253 and hence considered positive for CHIKV-specific antibodies. In addition, the presence of CHIKV nucleic acids was demonstrable in the muscle tissue by TaqMan real time PCR while, it was not detected in the blood and other tissues of the infected group of mice. All the control group of animals were negative for CHIKV nucleic acids.

**Fig 6:**
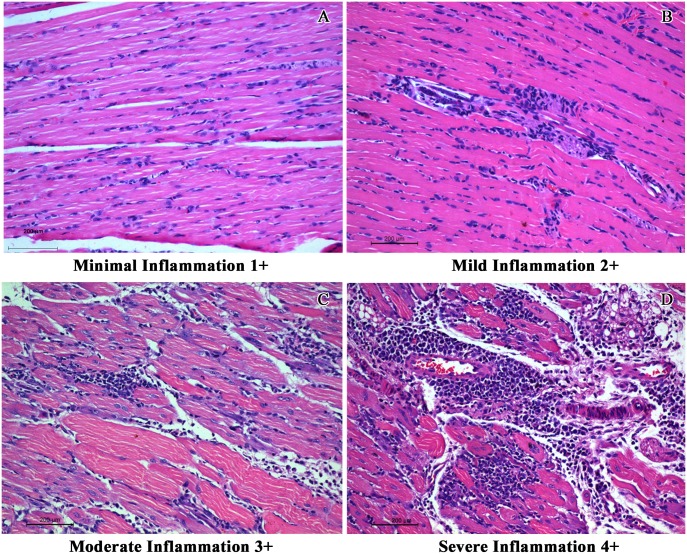
Representative images used for determining the overall inflammation score used in the study. Inflammation was scored in a scale of 1+ to 4+. A) Minimal inflammation (1+) characterized by sparse inflammatory infiltrate spreading along the interstitium and prominent muscle cell nuclei as a reparative response. B) Mild inflammation (2+) with lymphohistiocytic infiltration. C) Moderate inflammation (3+) with diffuse and focal aggregates of lymphohistiocytic cells spreading between the muscle fibres. Bluish muscle fibres in the section represents the regenerative muscle fibres in response to myositis. D) Severe inflammation (4+) with admixture of lymphohistiocytic and neutrophilic cell infiltrate with myonecrosis and regenerative activity of the muscle fibres. (Scale bar -200μM, H & E stain).

**Fig 7:**
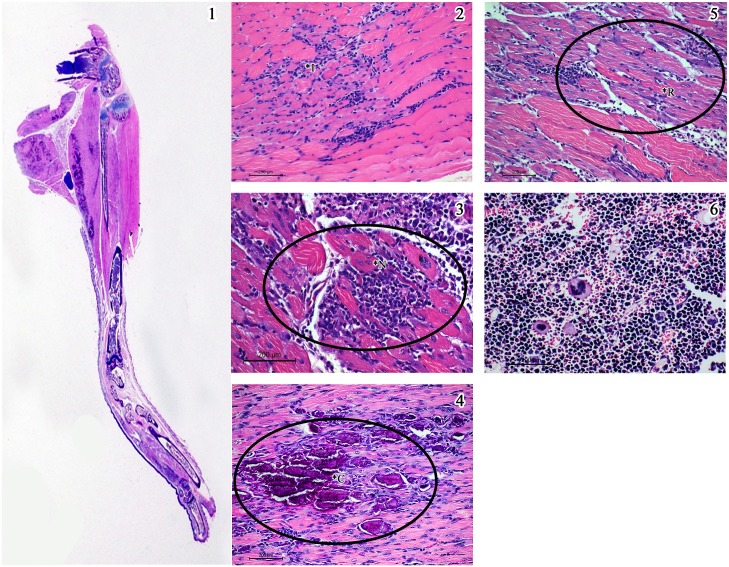
Salient histopathological features in the lower limb in mice injected with CHIKV (Group 1). 1) Whole mount section of the lower limb showing bluish patches of reactive calcification along the margins of the thigh muscle (x 2. H & E stain). 2) Reveals features of myositis with inflammatory infiltrate in Panel (*I). 3) Muscle necrosis with dense inflammatory infiltrate spreading in the endomysium (*N). 4) Mineralization of the necrosed tissue visible as dark blue flakes (*C). 5) Regeneration of the muscle fibres with the bluish pink colour (*R). 6) Hyperplasia of the bone marrow with prominent megakaryocytes. (Scale bar -200μM, H & E stain).

**Fig 8:**
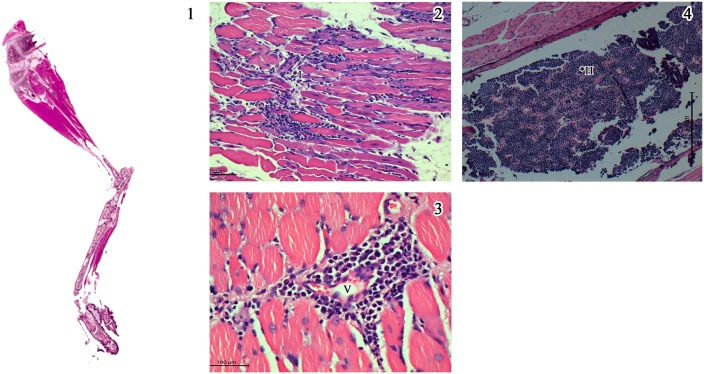
Salient histopathological features in lower limbs in mice injected with CHIKV specific peptides A & B (Group 4 & 5). 1) Whole mount section of lower limb of mice (x 2. H & E stain) with inflammation in the thigh muscle. 2) Endomysium is inflitrated by moderate degree of lymphohistiocytic cells (*I) and the muscle cells undergoing necrosis and atrophy. 3) Higher magnification revealing perivascular infiltration (*V) by lymphoplasmocytic cells and occasional polymorphs representing venulitis (Scale bar -100μM). 4) Marrow space of the femur filled with haemopoietic cells, suggesting marrow hyperplasia (*H). ((Scale bar -200μM, H & E stain).

**Fig 9:**
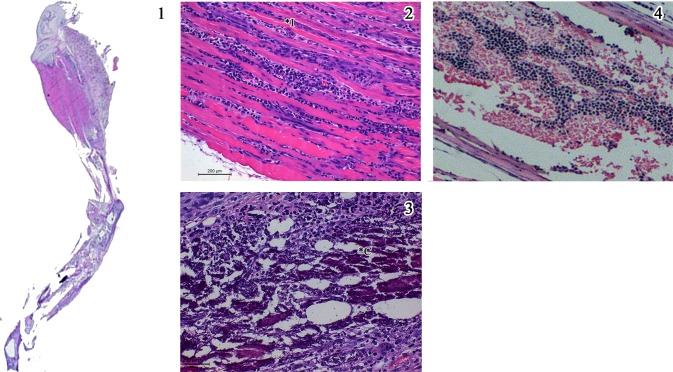
Salient histopathological features noted in H & E stained sections of lower limbs in mice injected with CHIKV followed by specific peptides A & B (Group 7 & 8). 1) Whole mount section of lower limb of the mice showing inflammatory infiltrate in the thigh muscle and extending along the tendon down (x 2. H & E stain). 2) Variable (dense at places) inflammation (*I) between the muscles fibres widening endomyseal space. Occasional bluish regenerating fibres are seen. 3) Dense reactive mineralisation of the necrotic fibres (*C) seen as bluish aggregates admixed with inflammation. 4) Marrow space of the thigh bone showing mild marrow haemopoietic cell hyperplasia. (Scale bar -200μM, H & E stain).

In order to have an objective assessment of the pathological features noted in all groups of animals, a semi quantitative scale for scoring the degree of inflammation centred mainly around the muscles was evolved and the slides were evaluated by a pathologist blinded to the groups and the same is depicted in Fig 6. The inflammation was graded as minimal (1+), mild (2+), moderate (3+) and severe (4+). The salient histopathological features noted in CHIKV infected mice were as follows: (i) the soft tissue around the elbow and knee joints were relatively normal with no evidence of synovitis or arthritis, while tissues close to the shoulder and the hip joint revealed moderate degree of lymphohistiocytic infiltration virus, (ii) the major muscles of the hip and the shoulder had multifocal lymphocytic infiltrate in the endomysium with myonecrosis, (iii) random and occasion muscle fibres close to inflammation revealed cytoplasmic basophilia and central nucleation with prominent nucleolus indicative of regenerative activity, similar to polymyositis noted in human subjects (iv) the synovium and periarticular soft tissue had sparse inflammation while the articular cartilage and the articular cavity were free of inflammation, (v) the epimysium around the muscle and tendinous portion close to the insertion had variable lympho-histiocytic inflammation indicating tenosynovitis, (vi) the striking pathology was mineralization of the necrosed muscle belly especially the lateral group of muscles close to the hip and shoulder joints similar to some cases of chronic polymyositis in human subjects. Other than these features noted in the limbs and joints all the other organs did not reveal any significant pathological changes. Some of the salient features noted in CHIKV infected mice (Group 1) are depicted in Fig 7. The degree of pathological damage centred on the muscles in the various groups of mice was graded and a comparative chart was prepared (Table 3). As evident from the table, the group of mice that were mock infected (Group 2) and subsequently did not receive any peptides revealed sparse (1+) inflammation in the muscles probably related to the procedure. Similar findings were also noted in the mock infected animals that received a single dose of Freund’s incomplete adjuvant (Group 6). In mice that received two doses of non-specific peptide but no virus (Group 5) hyperplasia of the bone marrow was noted with sparse inflammation (1+). On the other hand, mice that received two doses of CHIKV specific peptides but no virus (Groups 3 & 4) exhibited myositis, muscle necrosis, vasculitis and hyperplasia of the marrow (immune mediated inflammatory muscle and marrow reactive changes) and an overall inflammation score of 3+ (Fig 8). In the three groups of mice that received an initial inoculum of virus followed by a single dose of either CHIKV specific peptides (Groups 7&8) or non-specific peptide (Group 9) the pathological features were more florid (Fig 9). Amongst these three groups of animals, the mice that received virus followed by non-specific peptide exhibited features similar to those observed in mice that received virus alone (Group 1). The animals in Groups 7 and 8 had the highest overall inflammatory score (4+) and showed multiple features including myositis, muscle necrosis, focal regeneration, mineralization and calcification of the necrotic muscles, hyperplasia of marrow in the long bones.

**Table 3:** Compilation of salient features observed in histopathology among various experimental groups in the study and the overall inflammation score.

EXPERIMENTAL GROUPS	SALIENT FEATURES NOTED IN HISTOPATHOLOGY IN EACH EXPERIMENTAL GROUP						
	MYOSITIS	MUSCLE NECROSIS	MINERALISATION OF NECROSED MUSCLE	REGENERATION OF MUSCLES	VASCULITIS	HYPERPLASIA OF MARROW	OVERALL INFLAMMATION SCORE
**GROUP 1** **Virus control**	PRESENT	PRESENT	PRESENT	PRESENT	PRESENT	PRESENT	**3+**
**GROUP 2** **Mock infected**	ABSENT	ABSENT	ABSENT	ABSENT	ABSENT	ABSENT	**1 +**
**GROUP 3** **Peptide A control**	PRESENT	PRESENT	ABSENT	ABSENT	PRESENT	PRESENT	**3+**
**GROUP 4** **Peptide B control**	PRESENT	PRESENT	ABSENT	ABSENT	PRESENT	PRESENT	**3+**
**GROUP 5** **Non specific peptide control**	ABSENT	ABSENT	ABSENT	ABSENT	ABSENT	PRESENT	**1+**
**GROUP 6** **No peptide control**	ABSENT	ABSENT	ABSENT	ABSENT	ABSENT	ABSENT	**1+**
**GROUP 7** **CHIKV + Peptide A**	PRESENT	PRESENT	PRESENT	PRESENT	PRESENT	PRESENT	**4+**
**GROUP 8** **CHIKV+ Peptide B**	PRESENT	PRESENT	PRESENT	PRESENT	PRESENT	PRESENT	**4+**
**GROUP 9** **CHIKV + Non specific peptide**	PRESENT	PRESENT	PRESENT	ABSENT	PRESENT	PRESENT	**3+**

## Discussion

Molecular mimicry represents shared immunologic epitopes between a microbe and the host. In a viral system, viruses have been shown to have cross reactive epitopes with host self proteins [11]. Molecular mimicry can be either in the form of sequence homology wherein the host and the infectious agent share the sequence of similar or identical amino acids or it might be due to the conformational similarity between the host and the infectious agent in question [11]. Molecular mimicry is one of the major mechanisms for the induction of autoimmune diseases through the activation of autoreactive T cells in the host immune system. Several elegant examples of molecular mimicry leading to autoimmune manifestations have been described following bacterial and viral infections [11].

Chikungunya fever is a self limiting illness, however in 20–30% of the patients arthralgia persists for a period of two years and above [12]. More than half of all CHIKV infected patients in La Reunion Island during the 2005–2006 epidemics had complaints of persistent joint pain / recurring stiffness [13]. The arthritis attributed to CHIKV infection indeed mimics rheumatoid arthritis, as discussed by Bouquillard et al [14] wherein 21 patients infected with CHIKV in Reunion islands developed RA. Further, Malvy et al [15] suggested that molecular mimicry may be responsible for chronic manifestations as symptoms continue to persist despite CHIKV not being detectable in the synovial tissue. We investigated molecular mimicry in this study by using a combined approach of identifying homologous regions between CHIKV glycoprotein E1 protein and host tissue components using bioinformatics tools, the ability of these designed peptides to cross react with serum samples from CHIKV infected patients and inducing immune mediated joint and muscle pathology in a mouse model.

In order to determine if there are any “*arthritogenic*” motifs within the E 1 protein, a multiple sequence alignment of amino acid sequences of E1 glycoprotein of CHIKV was carried out with other alphaviruses such as ONNV, RRV, SFV and EEEV using CLUSTALW (Fig 2). The alignment revealed that two common motif(s) SKD and KCA were present only in arthritogenic alphaviruses such as CHIKV, RRV and ONNV but not in the *‘encephalitogenic’* alphaviruses such as SFV and VEEV (Fig 2). The presence of these two motifs seen only in *arthritogenic* alphaviruses lead us to postulate that these two motifs may have a role in the development of arthralgia which is a hallmark in the disease produced by these group of viruses. Simultaneously, the immunodominant epitopes in the CHIKV E1 glycoprotein were deduced using IEDB and EMBOSS programs which use criteria like presence of beta turn, surface accessibility, flexibility, antigenicity and hydrophilicity of the regions to be studied. On combining the common results obtained with these two programs, four immunodominant regions were obtained in CHIKV E1 glycoprotein (Peptide A, B, C and D). Interestingly, it was observed that the “*arthritogenic*” motifs SKD and KCA were also present in the immunodominant Peptides A and B respectively.

Sequence homology comparisons between CHIKV E1 glycoprotein and various human proteins using BLAST revealed that a homology of four consecutive amino acids TQLV/TELV exist between the CHIKV E1 glycoprotein and HLA-B27 molecule (Fig 3). It is a well established that HLA B27 has been implicated in the pathogenesis of autoimmune arthritis and ankylosing spondylitis [16]. Interestingly this consecutive sequence of amino acids was also present in one of the immunodominant peptides (Peptide A) of CHIKV E1 glycoprotein (Fig 3). Having ascertained that there is amino acid homology between CHIKV E1 glycoprotein and HLA B27 molecule, the occurrence of structural homology was also explored using the BioXGEM program. This program searches for the longest common substructures existing between the query structure and every structure in the database. The output of the query while executing the program was however limited to the first 100 hits. Amongst these 100 proteins, further analysis was restricted only to human proteins present in the output. Amongst the 20 human proteins in the list (Table 2), six proteins were shortlisted as they are known to contribute to either inflammation or arthritic pathology. The most prominent amongst them was complement components C3and C5. Indeed, C3 complement component has been implicated in inflammation and tissue injury in other related alphaviral infections [17] and therefore further analysis was confined to the homology between the CHIKV E1 glycoprotein and the C3 complement component. The analysis showed that the homology occurred between Von Willebrand Factor (VWF) domain of C3 and CHIKV E1 glycoprotein (Panel A, Fig 5). Interestingly, the amino acid sequences in the region of CHIKV E1 glycoprotein exhibiting homology were also present in the immunodominant Peptides A and B (Panel B, Fig 5). In summary, combining the data from the various bioinformatics approaches and employing a logical algorithm relevant to the pathogenesis of arthritis the choice narrowed down to two immunodominant peptides of CHIKV E protein (Peptides A & B).Therefore all further experiments were carried out using only these two peptides.The Peptides A and B were used in an ELISA to assess the immunoreactivity of serum samples obtained from CHIKV confirmed patients as well as healthy control subjects. The serum samples obtained from CHIKV confirmed patients (n = 36) showed reactivity to both the peptides to varying degrees. Antibodies to Peptide A was noted in 24/36 (66.66%) of samples while 27/36 (75%) of serum samples showed reactivity to Peptide B (Fig 5). These results indicate that the two peptides are indeed being recognized by the host immune system during CHIKV infection. As evident form Fig 5, it was interesting to note that the sera from three patients who had persistent arthralgia 12 weeks after the onset of initial symptoms indeed exhibited much higher OD values (0.9 to 1.3 for peptide A and 1.2 for peptide B) to the two peptides A & B as compared to other patients (OD values were close to cut off and between 0.4 and 0.6). Although, a quantitative ELISA would have delineated these differences better our ELISA was designed only as qualitative assay.

Further, experiments were conducted to investigate if peptide A and peptide B were capable of inducing pathology in an experimental mouse model. The results indicated that these two peptides on their own were able to induce significant inflammation in the muscles of C57BL/6J mice (Fig 8 and Table 3) similar to that observed in animals (3+) that were injected with CHIKV. Further, animals that were primed initially with CHIKV followed by a subsequent injection of the two CHIKV peptides exhibited enhanced inflammatory pathology (4+) as compared to animals that were injected with peptides or virus alone (Fig 9 and Table 3). On the contrary, animals that received an unrelated peptide (i.e. not containing the arthritogenic motifs or exhibiting homology to host proteins) either before or after priming with CHIKV exhibited minimal muscle inflammation (1+). Collectively these observations validate the hypothesis that molecular mimicry between CHIKV E1 protein and host proteins does contribute to pathology in CHIKV infection. Such observations have not been reported hitherto in CHIKV infection although molecular mimicry as a mechanism leading to autoimmune phenomena has been demonstrated in several microbial infections including viruses and bacteria [18]. Among the viruses, molecular mimicry has been noted in Theiler murine encephalitis virus (TMEV), Hepatitis B virus (HBV), and SFV and Coxsackie viral infections [11]. In SFV infection of C57BL/6J mice a similar approach using bioinformatic tools derived peptides and validation of these peptides *invivo* for their ability to induce autoimmune demyelination was undertaken [19]. An algorithmic approach was used to demonstrate amino acid homology between immunogenic epitopes of SFV and various myelin proteins. The criterion used for the occurrence of molecular mimicry was the presence of three similar consecutive amino acids. It was observed that myelin oligodendrocyte protein (MOG) shared homology with SFV E2 glycoprotein. The injection of a peptide containing the sequence of shared amino acids into mice caused demyelination with presence of multifocal vacuolation in the CNS white matter [19]. The phenomenon of molecular mimicry has also been studied in SARS infection [20]. Eleven peptides derived from SARS spike(S) protein which shared homology with various human proteins were synthesised and their reactivity was assessed using serum samples obtained from SARS patients. Serum samples recognised only 2/11 peptides and the authors concluded that these two peptides may contribute to viral pathogenesis through the phenomena of molecular mimicry.

The limitations of the present study are twofold. Firstly, HLA-B27 typing was not performed in the CHIKV confirmed patients and controls of this study. However, serum reactivity of Peptide A which shared homology with HLA B27 molecule suggests that this molecule may have an important role to play in persistent arthralgia as 3/36 patients exhibited high reactivity to it. The geographical and ethnic variation in the prevalence of HLA-B27 globally is well documented [21] and further its occurrence in CHIKV related complications is also reported to be very low [15, 22]. Therefore it may be argued that the observance of molecular mimicry between CHIV E1 glycoprotein and HLA-B27 may not be the major factor contributing to complications that ensue following acute infection. Secondly, the time interval between the injection of CHIKV and the peptides into animal the model (Groups 7& 8) is rather short rendering it difficult to conclude whether the inflammatory response noted was due to the immunopathologic process of molecular mimicry or a direct inflammatory effect of both the virus and the peptide. However, the muscle fibrosis and calcification in the muscles noted in the experimental animals recapitulates immune mediated polymyositis in humans during the evolution and progression. The other limitation with all studies conducted using animal models to demonstrate molecular mimicry as a mechanism of pathogenesis is that an adjuvant such as CFA/FICA is invariably required to elicit immune mediated damage. This suggests that in addition to having cross reacting disease inducing epitopes, sufficient activation of antigen presenting cells is required. Consequently, the most difficult part in the studies investigating molecular mimicry is to correlate/extrapolate the findings obtained *in vitro* and/or animal studies to the disease occurring in the natural host. Despite this limitation, the concept of molecular mimicry remains a viable hypothesis for framing questions and approaches to understanding the pathogenetic mechanisms involved during the disease process. Therefore, it would be definitely worthwhile to pursue future studies with the two CHIKV peptides described in this study using transgenic animal models and human T and B cell responses to the peptides.
